# Comparative effectiveness and safety of preventive treatments for vestibular migraine: a systematic review and network meta-analysis

**DOI:** 10.1186/s12883-025-04490-0

**Published:** 2025-12-30

**Authors:** Sindhu Vasireddy, Shankar Biswas, Raja Kollu, Elangovan Krishnan, Mohammed Semaal Khan, Fasil C, Arjun Jayakumar, Reena Acharya

**Affiliations:** 1 Specialist Neurology, Department of Neurology, NMC Specialty Hospital, Abu Dhabi, United Arab Emirates; 2 Department of Internal Medicine, Ivano-Frankivsk National Medical University, Ivano-Frankivsk, Ukraine; 3 Specialist Radiology, Department of Radiology, NMC Specialty Hospital, Abu Dhabi, United Arab Emirates; 4 Department of Internal Medicine, Madras medical college, Chennai, Tamil Nadu India; 5 Department of Internal Medicine, Kerala University of Health Sciences, Thrissur, Kerala India; 6 Department of Internal Medicine, Sri Ramchandra Medical College, Chennai, Tamil Nadu India; 7 Department of Internal Medicine, GCS Medical College and Research Centre, Ahmedabad, Gujarat India

**Keywords:** Vestibular migraine, Network meta-analysis, Prevention, Prophylaxis, CGRP, Systematic review

## Abstract

**Background:**

Vestibular migraine causes recurrent vertigo attacks that significantly impact quality of life. While various preventive medications are used, their comparative effectiveness was unknown. Previous systematic reviews have been limited by pairwise comparisons only or exclusion of newer treatments. The lack of head-to-head trials comparing all available treatments further complicates evidence-based decision-making. This evidence gap has real-world consequences, has also substantial economic burden of Vestibular migraine. There is a need for direct comparison studies between the most promising treatments to provide clearer guidance for clinical practice.

**Objective:**

To determine the comparative effectiveness and safety of preventive treatments for vestibular migraine through systematic review and network meta-analysis. Given the lack of head-to-head randomized trials, a network meta-analysis (NMA) provides the most appropriate method to compare available treatments by combining both direct and indirect evidence.

**Methods:**

We searched Embase, Scopus, PubMed, and Cochrane Library from inception to January 15, 2025. We included randomized controlled trials (RCTs) and prospective observational studies (*n* ≥ 30 for CGRP antagonists) comparing preventive treatments for vestibular migraine diagnosed according to either Bárány Society/International Headache Society criteria (post-2012) or Neuhauser criteria (pre-2012). Primary outcomes were monthly vertigo frequency and quality of life (DHI scores). We conducted frequentist network meta-analysis and assessed certainty using GRADE.

**Results:**

From 340 identified records, nine studies met inclusion criteria. Five RCTs (419 patients) comparing seven treatments were included in the network meta-analysis. All treatments significantly reduced monthly vertigo attacks versus control. Propranolol ranked highest (P-score: 0.794; -7.04 attacks/month, 95% CI -12.77 to -1.31), followed by valproic acid (-5.95, 95% CI -9.01 to -2.89) and venlafaxine (-5.94, 95% CI -8.98 to -2.90). Galcanezumab showed moderate efficacy (-5.80, 95% CI -10.61 to -0.99) with zero discontinuations. Network heterogeneity was negligible (τ²<0.001). Evidence certainty was moderate for galcanezumab and low to very low for other treatments.

**Conclusions:**

All evaluated treatments effectively reduce vertigo frequency in vestibular migraine. While propranolol showed the largest effect, this relied on indirect evidence. Galcanezumab offers the best balance of efficacy, tolerability, and evidence quality. Head-to-head trials are urgently needed.

**PROSPERO registration:**

CRD420251089507.

**Graphical Abstract:**

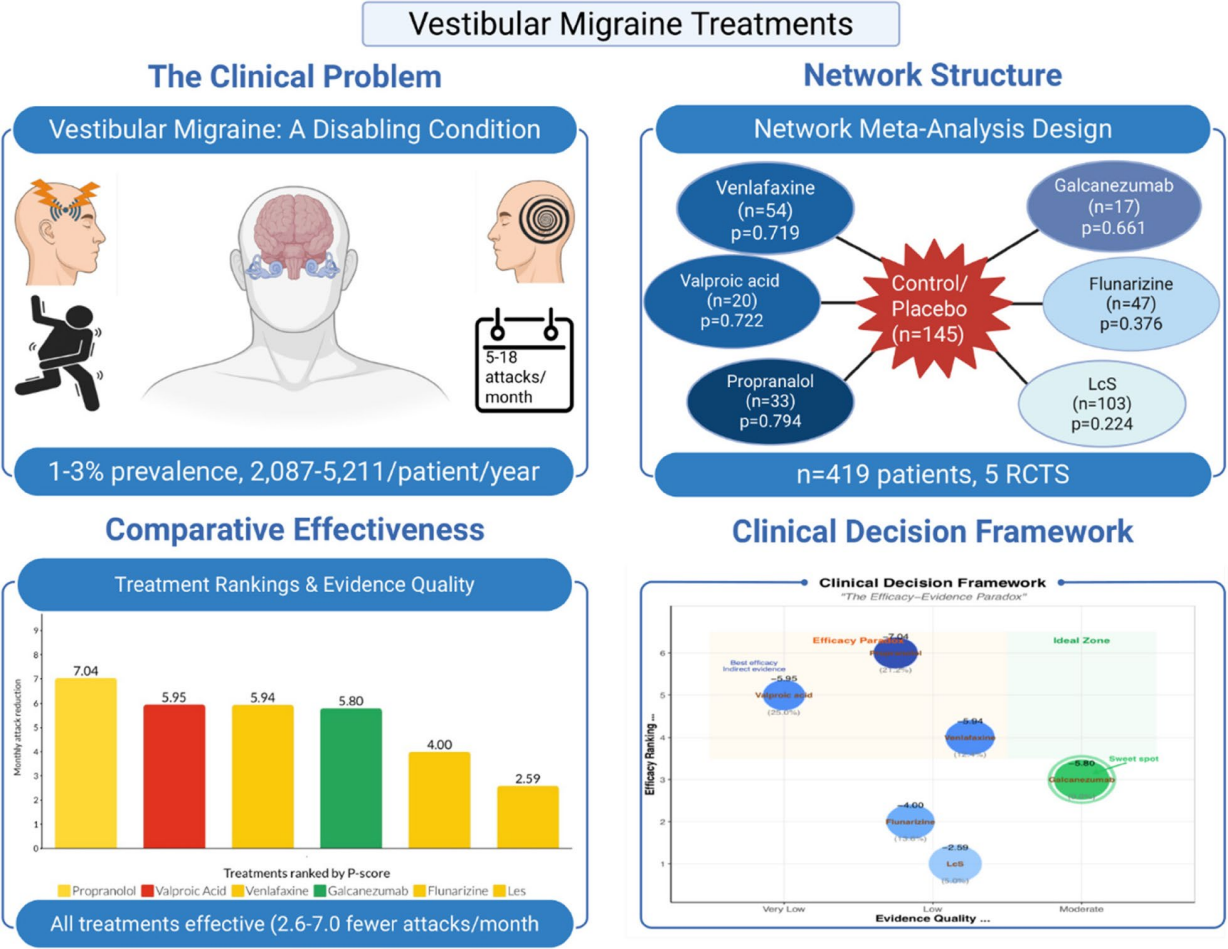

**Supplementary Information:**

The online version contains supplementary material available at 10.1186/s12883-025-04490-0.

## Author summary

Vestibular migraine causes recurrent vertigo attacks that significantly impact quality of life. While various preventive medications are used, their comparative effectiveness was unknown. We analyzed all available clinical trials comparing preventive treatments using network meta-analysis methods that allow indirect comparisons between treatments never studied head-to-head. We found that all medications significantly reduced vertigo attacks, with traditional migraine preventives like propranolol showing the largest benefits. However, the newer CGRP antibody galcanezumab had the strongest evidence quality and best tolerability. Our analysis helps clinicians and patients choose between treatments by balancing effectiveness with evidence quality and side effects. The findings highlight the need for direct comparison studies between the most promising treatments to provide clearer guidance for clinical practice.

## Introduction

Vestibular migraine (VM) affects 1–3% of the general population and represents a leading cause of recurrent vertigo [1]. Despite its substantial impact on quality of life and healthcare utilization, evidence-based treatment guidance remains limited [2, 3]. The management of VM presents unique challenges, as patients experience both vestibular and migrainous symptoms that significantly impair daily functioning.

Current treatment approaches are largely empirical, borrowing from migraine prophylaxis without VM-specific evidence. Traditional preventive medications include beta-blockers, calcium channel blockers, antidepressants, and anticonvulsants [4, 5]. Recently, calcitonin gene-related peptide (CGRP) has been implicated in vestibular sensitization in migraine models, suggesting CGRP monoclonal antibodies as potential treatments [6]. However, the comparative effectiveness of these diverse treatment options remains unclear, creating uncertainty for clinicians and patients.

Previous systematic reviews have been limited by pairwise comparisons only or exclusion of newer treatments [7, 8]. The lack of head-to-head trials comparing all available treatments further complicates evidence-based decision-making. This evidence gap has real-world consequences, as shown by the substantial economic burden of VM, with annual incremental costs of $2,087-$5,211 per patient and societal costs reaching $60 billion [3]. The most recent network meta-analysis by Chen et al. (2023) included seven treatments across 828 patients but did not evaluate CGRP antagonists, which have emerged as promising options given the role of CGRP in vestibular sensitization [9]. This evidence gap is particularly relevant as CGRP antibodies enter clinical practice.

Network meta-analysis enables simultaneous comparison of multiple treatments using both direct and indirect evidence, providing a comprehensive evidence synthesis for clinical decision-making [10]. This approach is particularly valuable in VM, where the limited number of trials makes traditional pairwise meta-analysis inadequate. We conducted this systematic review and network meta-analysis to determine the comparative effectiveness and safety of all available preventive treatments for vestibular migraine.

## Methods

### Protocol and registration

This systematic review followed a pre-specified protocol registered with PROSPERO CRD420251089507 and reported according to PRISMA-NMA guidelines [11]. As this study involved analysis of published aggregate data only, ethics committee approval was not required.

### Eligibility criteria

We included studies meeting the following criteria:

#### Population

Adults (≥ 18 years) with vestibular migraine diagnosed according to Bárány Society/International Headache Society criteria [12] or Neuhauser criteria (for pre-2012 studies) [13]. Because diagnostic definitions evolved over time, we included studies using Neuhauser’s criteria prior to 2012 and Bárány Society/IHS criteria thereafter. This variation may introduce bias, which we address in the Discussion.

#### Interventions

Any pharmacological preventive treatment administered for ≥ 12 weeks, including beta-blockers, calcium channel blockers, antidepressants, anticonvulsants, CGRP antagonists, and other preventives.

#### Comparators

Placebo, active comparators, or usual care.

#### Outcomes

Primary outcomes were (1) change in monthly vertigo frequency/days and (2) quality of life measured by Dizziness Handicap Inventory (DHI). Secondary outcomes included treatment discontinuation, responder rates (≥ 50% reduction), and migraine headache frequency.

#### Study design

Randomized controlled trials and prospective observational studies (minimum *n* = 30 for CGRP antagonists given limited RCT evidence).

### Information sources and search strategy

We searched Embase (Ovid), Scopus, PubMed, and Cochrane Library from inception to January 15, 2025, without language restrictions. The search strategy combined terms for vestibular migraine, prevention, and study design (Supplementary Table 1). We additionally searched ClinicalTrials.gov, WHO ICTRP, and reference lists of included studies.

### Study selection and data extraction

Two reviewers independently screened titles/abstracts and full texts using Covidence. Discrepancies were resolved through discussion or third reviewer consultation. We extracted study characteristics, participant demographics, intervention details, and outcome data using standardized forms. For missing data, we contacted authors or used established imputation methods [14].

### Risk of bias assessment

We assessed risk of bias using the Cochrane Risk of Bias 2.0 tool for RCTs [15] and ROBINS-I for observational studies [16]. Assessments covered randomization, deviations from intended interventions, missing data, outcome measurement, and selective reporting.

### Data synthesis

#### Pairwise meta-analysis

We calculated mean differences (MD) for continuous outcomes using random-effects models. For studies reporting only endpoint values, we calculated change scores using a correlation coefficient of 0.5 [14].

#### Network meta-analysis

We performed frequentist network meta-analysis using the netmeta package (version 3.2-0.2.2) in R [17]. We assumed common heterogeneity across comparisons and used restricted maximum likelihood estimation. Treatment rankings were calculated using P-scores [18].

#### Assessment of inconsistency

We planned to assess consistency using the design-by-treatment interaction model [19] and node-splitting, [20] but no closed loops existed in our network.

#### Certainty of evidence

We assessed certainty using GRADE for network meta-analysis, [21] considering risk of bias, inconsistency, indirectness, imprecision, and publication bias. We started at high certainty for RCTs and downgraded based on identified limitations.

#### Sensitivity and additional analyses

We conducted pre-specified sensitivity analyses: (1) fixed-effect model, (2) inclusion of observational studies, (3) exclusion of high risk of bias studies. We performed threshold analysis to assess robustness [22] and examined drug class effects.

## Results

### Study selection

**Fig. 1:**
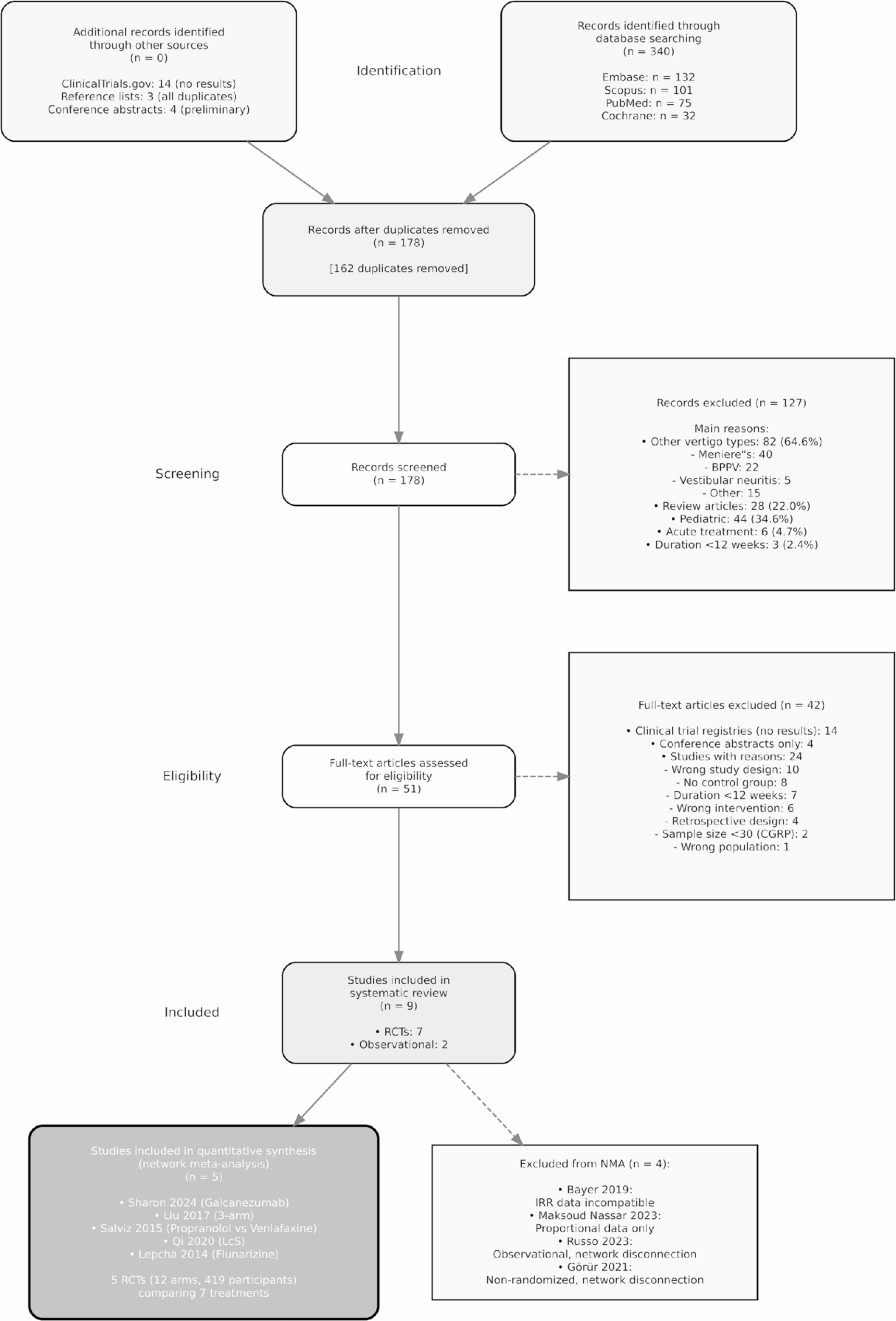
PRISMA flow diagram study selection process showing identification, screening, and inclusion of studies for systematic review and network meta-analysis

Database searches identified 340 records. After removing 162 duplicates, we screened 178 records and excluded 127 based on title/abstract. Of 51 full-text articles assessed, we excluded 44 (Supplementary Table 2), leaving nine studies for inclusion (Fig. 1). Five RCTs were included in the network meta-analysis.

### Study characteristics

The nine included studies comprised seven RCTs and two observational studies, enrolling 588 total participants (Table 1). Five RCTs (*n* = 419) formed a connected network for meta-analysis: Sharon 2024 (galcanezumab vs. placebo), [23] Liu 2017 (three-arm comparing flunarizine, venlafaxine, valproic acid), [24] Salviz 2015 (propranolol vs. venlafaxine), [25] Qi 2020 (Lactobacillus casei Shirota (LcS) vs. symptomatic control), [26] and Lepcha 2014 (flunarizine vs. control) [27].

**Table 1:** Characteristics of included studies

Study	Country	Design	*N*	Interventions	Duration	Primary Outcome	Risk of Bias
Sharon 2024 [23]	USA	RCT, DB	40	Galcanezumab vs. placebo	12 weeks	VM-PATHI	Low
Liu 2017 [25]	China	RCT, SB	75	Flunarizine vs. venlafaxine vs. valproic acid	12 weeks	Vertigo frequency	High
Salviz 2015	Turkey	RCT, OL	64	Propranolol vs. venlafaxine	12 weeks	Vertigo attacks	Some concerns
Qi 2020 [26]	China	RCT, DB	247	LcS vs. control	12 weeks	Vertigo frequency	Some concerns
Lepcha 2014 [27]	India	RCT, OL	52	Flunarizine vs. control	12 weeks	Vertigo frequency	Some concerns
Bayer 2019 [28]	Germany	RCT, DB	130	Metoprolol vs. placebo	24 weeks	Vertigo attacks	High
Maksoud Nassar 2023 [29]	Egypt	RCT, OL	45	Cinnarizine vs. propranolol vs. topiramate	12 weeks	Vertigo frequency	High
Russo 2023 [30]	Italy	Prospective	50	CGRP mAbs	≤ 18 months	Vertigo days	Moderate
Görür 2021 [31]	Turkey	Non-RCT	60	Oral meds ± BTX	12 weeks	DHI	Serious

Two RCTs could not be included: Bayer 2019 reported only incidence rate ratios, [28] and Maksoud Nassar 2023 provided only proportional changes [29]. Two observational studies (Russo 2023, [30] Görür 2021 [31]) were analyzed separately.

### Patient characteristics

Participants were predominantly female (60–94%) with mean ages 40–53 years (Table 2). Baseline vertigo frequency ranged from 5 to 18 attacks/month. Most studies (5/9) used Bárány Society/IHS diagnostic criteria. Mean disease duration and previous treatment details were incompletely reported.

**Table 2:** Network meta-analysis results: treatment effects vs. control

Treatment	MD (95% CI)	*P*-value	*P*-score	Rank	GRADE Certainty
Propranolol	−7.04 (−12.77 to −1.31)	0.016	0.794	1	Low
Valproic acid	−5.95 (−9.01 to −2.89)	< 0.001	0.722	2	Very low
Venlafaxine	−5.94 (−8.98 to −2.90)	< 0.001	0.719	3	Low
Galcanezumab	−5.80 (−10.61 to −0.99)	0.018	0.661	4	Moderate
Flunarizine	−4.00 (−6.54 to −1.46)	0.002	0.376	5	Low
LcS	−2.59 (−3.31 to −1.87)	< 0.001	0.224	6	Low

*MD* Mean difference in monthly vertigo attacks, *CI* Confidence interval

### Risk of bias

Only Sharon 2024 [23] achieved low risk of bias across all domains. Three RCTs had some concerns primarily due to open-label design or incomplete outcome data. Liu 2017 [24] and Bayer 2019 [28] were high risk due to deviations from intended interventions and missing outcome data, respectively (Supplementary Fig. 1; detailed assessment in Supplementary Table 3).

### Network structure

**Fig. 2:**
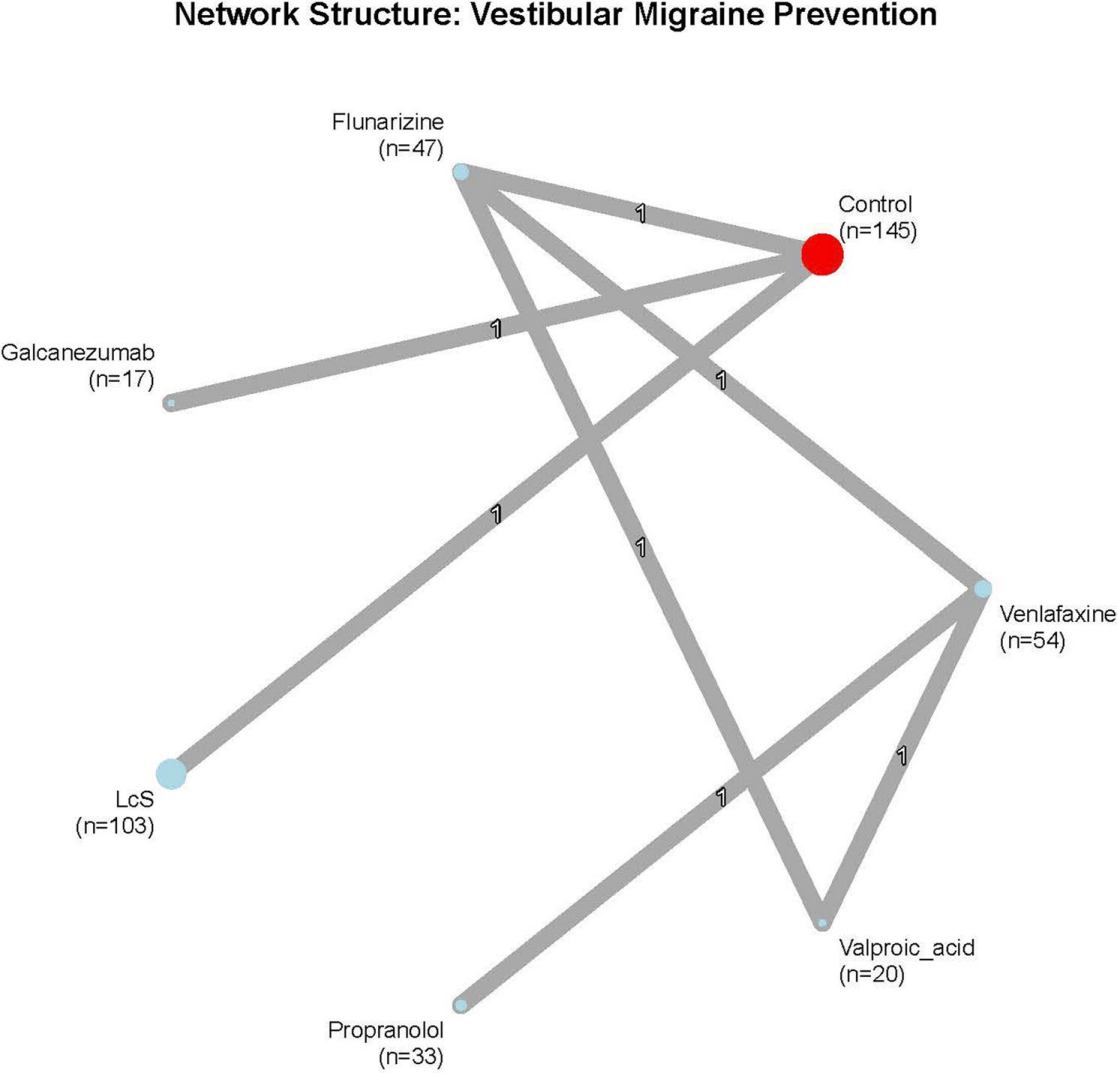
Network plot network structure showing direct comparisons between treatments. Node size proportional to total sample size; edge thickness proportional to number of studies. Control (red), active treatments (blue)

The network included seven treatments across five trials forming a star-shaped configuration with no closed loops (Fig. 2). Three treatments (galcanezumab, LcS, flunarizine) had direct placebo comparisons. Propranolol and valproic acid effects relied entirely on indirect evidence through venlafaxine connections.

### Primary outcome: monthly vertigo frequency

**Fig. 3:**
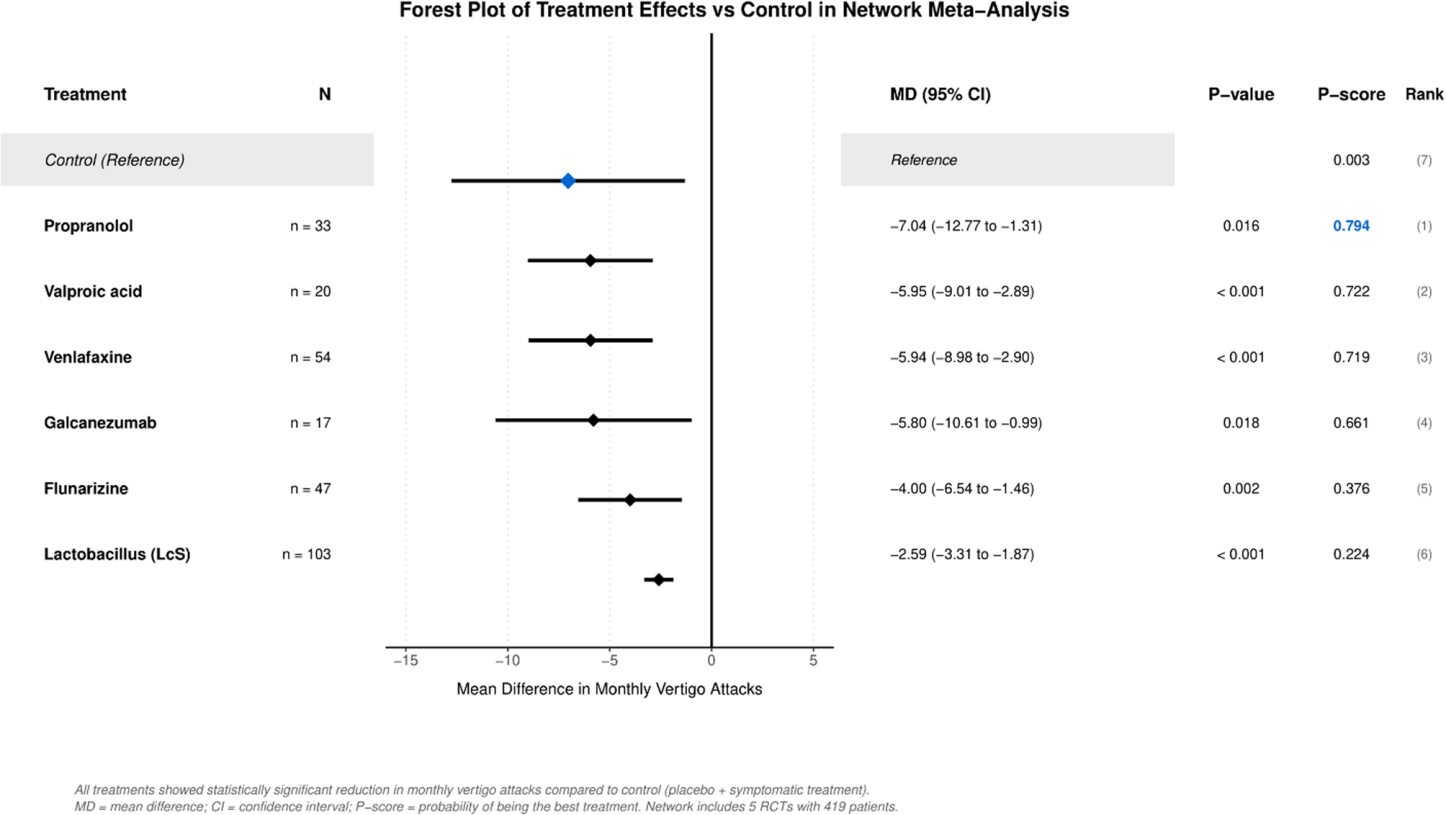
Forest plot of treatment effects vs. control mean differences in monthly vertigo attacks with 95% confidence intervals. All treatments showed statistically significant reduction compared to control (placebo/symptomatic treatment)

**Fig. 4:**
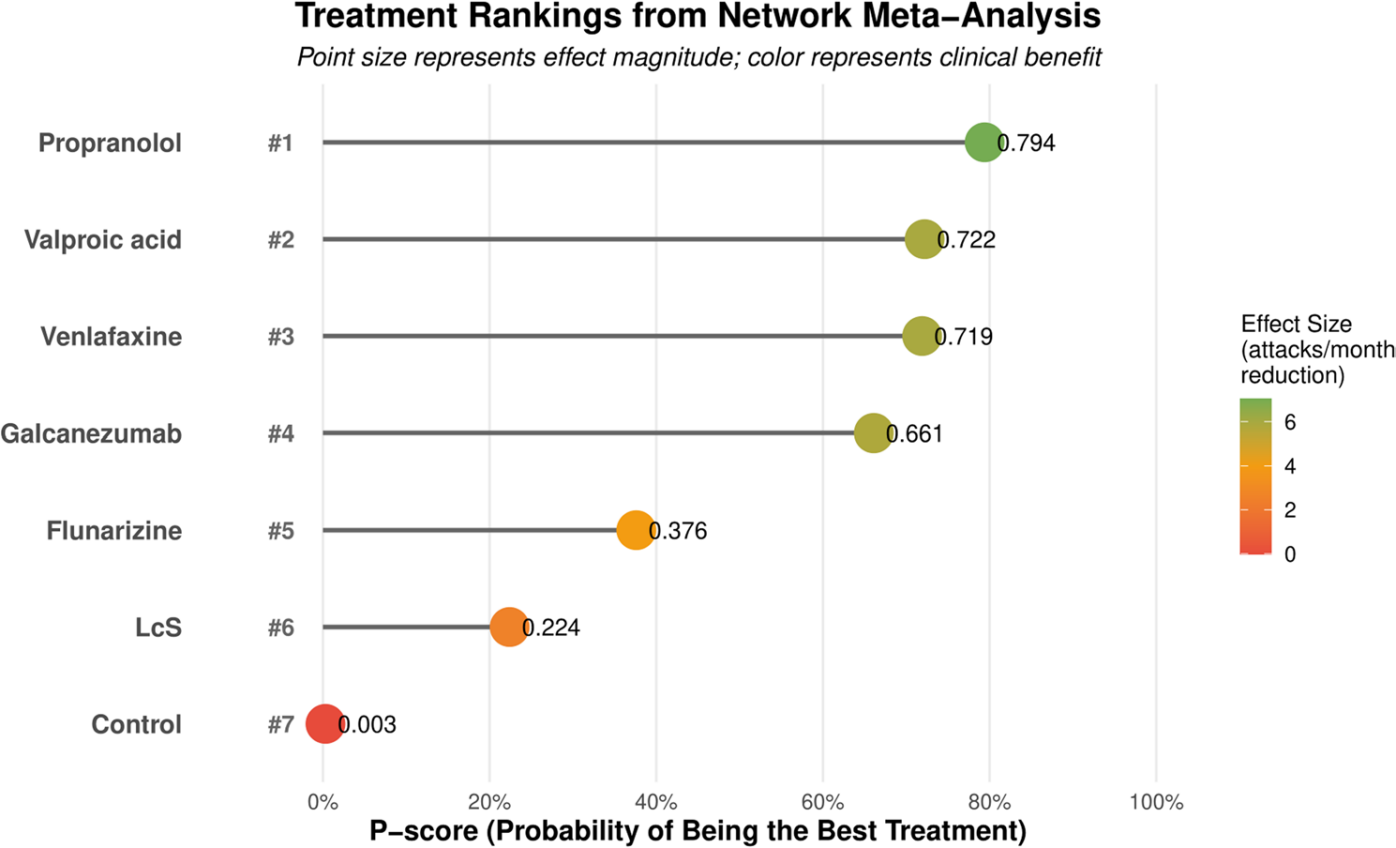
Treatment rankings based on P-scores ranking of treatments from highest (1) to lowest (7) probability of being the best treatment. Point size represents effect magnitude; color gradient indicates clinical benefit

All treatments significantly reduced monthly vertigo attacks compared to control (Fig. 3; Table 2). Treatment effects ranged from − 2.59 (95% CI −3.31 to −1.87) for LcS to −7.04 (95% CI −12.77 to −1.31) for propranolol. Network heterogeneity was essentially zero (Q = 1.93 × 10⁻³¹, df = 0, τ²<0.001), indicating consistent treatment effects. Treatment rankings were calculated using P-scores (Table 3; Fig. 4).

**Table 3:** Network meta − analysis results: ranked by P − score

Rank	Treatment	MD (95% CI)	*P* − value	*P* − score
1	Propranolol	−7.04 (− 12.77 to − 1.31)	0.016 *	0.794
2	Valproic acid	−5.95 (− 9.01 to − 2.89)	0.000 *	0.722
3	Venlafaxine	−5.94 (− 8.98 to − 2.9)	0.000 *	0.719
4	Galcanezumab	−5.8 (− 10.61 to − 0.99)	0.018 *	0.661
5	Flunarizine	−4 (− 6.54 to − 1.46)	0.002 *	0.376
6	LcS	−2.59 (− 3.31 to − 1.87)	0.000 *	0.224

*Statistically significant at *p* < 0.05

### Secondary outcomes

#### Quality of life (DHI Scores)

Four studies reported DHI outcomes. Galcanezumab showed the largest improvement (−22.0 points vs. −8.3 placebo, *p* = 0.017) [23]. Venlafaxine effects varied across studies (−10.4 to −31.0 points). Network connectivity was insufficient for DHI network meta-analysis.

#### Responder rates

Three studies reported ≥ 50% responder rates: propranolol 88%, [25] venlafaxine 85%, [25] galcanezumab 77% vs. placebo 48% [23]. Complete vertigo control ranged from 18 to 50% with active treatments vs. 10% placebo.

#### Safety and tolerability

Discontinuation rates varied significantly: galcanezumab 0%, [23] venlafaxine 8.7–16.1%, [24, 25] flunarizine 13.6%, [27] propranolol 21.2%, [25] valproic acid 25% [24]. Although flunarizine showed significant short-term efficacy, its use is restricted or withdrawn in several countries due to concerns about long-term adverse effects such as depression and parkinsonism. The relatively favorable safety profile observed in our included trials likely reflects short treatment durations. Common adverse events included hypotension/bradycardia (beta-blockers), weight gain (flunarizine), and nausea (multiple classes).

#### Certainty of evidence

GRADE assessment revealed moderate certainty only for galcanezumab (direct placebo comparison, low risk of bias). Other treatments had low or very low certainty due to indirectness (indirect comparisons only) and risk of bias (Table 3).

#### Sensitivity analyses

Fixed-effect analysis yielded identical results to random-effects (maximum difference 0.003 attacks/month), confirming negligible heterogeneity. Inclusion of observational studies resulted in a disconnected network, preventing integration. Low risk of bias analysis was unfeasible with only one qualifying study.

#### Threshold analysis

Propranolol’s top ranking proved relatively robust, requiring a 15.5% reduction (1.09 attacks/month) to fall below valproic acid. Galcanezumab would need a 21.4% improvement to overtake propranolol. All treatments except LcS exceeded the clinical significance threshold of 2 attacks/month reduction by substantial margins.

#### Drug class analysis

Each pharmacological class was represented by single agents, preventing formal class effect estimation. Traditional preventives (beta-blockers, anticonvulsants, antidepressants, calcium channel blockers) showed numerically larger effects (mean − 5.73 attacks/month) than novel approaches (CGRP antagonist, probiotic; mean − 4.20 attacks/month).

## Discussion

### Principal findings

This network meta-analysis provides the most comprehensive evidence synthesis for vestibular migraine prevention to date. All evaluated treatments significantly reduced vertigo frequency, with traditional preventives showing numerically larger effects than novel treatments. However, evidence quality varied dramatically, creating an efficacy-evidence paradox where the statistically top-ranked treatment (propranolol) relied entirely on indirect evidence while the best-evidenced treatment (galcanezumab) ranked fourth. It is important to note that propranolol’s effect estimate was derived exclusively from indirect evidence, meaning its comparison relied on shared placebo or active comparator arms rather than direct head-to-head trials. In NMA, indirect evidence allows estimation of relative effects when two treatments have not been directly compared in a single trial.

### Comparison with existing literature

Our findings both confirm and extend previous systematic reviews of vestibular migraine prevention. Two previous pairwise meta-analyses [7, 32] demonstrated efficacy of traditional preventives but were limited to direct comparisons only. Chu et al. (2023) conducted a network meta-analysis but excluded CGRP antagonists and included only four treatments [33]. Our findings extend those of Chen et al. (CNS Drugs 2023), [9] who conducted an earlier NMA of vestibular migraine preventives but did not include CGRP antagonists. While Chen et al. included 7 RCTs with 828 participants, our analysis included 5 RCTs with 419 participants due to stricter methodological criteria. Specifically, we required all studies to have ≥ 30 participants and excluded Bayer 2019 (metoprolol, *n* = 130) due to incompatible outcome reporting (incidence rate ratios rather than mean differences). We focused on studies reporting monthly vertigo frequency as the primary outcome to ensure network consistency. This methodological rigor resulted in zero heterogeneity (τ²<0.001), suggesting our effect estimates may be more reliable despite the smaller sample size. Most importantly, our inclusion of galcanezumab provides the first network meta-analysis evidence for CGRP antagonists in vestibular migraine, addressing a critical gap as these agents enter clinical practice. Our inclusion of galcanezumab represents the first network meta-analysis evidence for CGRP antagonists in vestibular migraine.

The magnitude of effects we observed (−2.6 to −7.0 attacks/month) aligns with the 50–75% reduction reported in previous reviews [31]. However, our finding of zero heterogeneity contrasts with moderate heterogeneity (I²=40–60%) in previous analyses, likely reflecting our stricter inclusion criteria and standardized outcome timing [32]. The high placebo response we observed (30–48%) is consistent with vestibular migraine trials generallyand emphasizes the importance of controlled study designs [34].

### Clinical implications

Treatment selection should balance statistical rankings with evidence quality and patient factors. For patients prioritizing efficacy with acceptable tolerability, propranolol remains a reasonable first choice despite indirect evidence. For those prioritizing evidence quality and tolerability, galcanezumab offers advantages with zero discontinuations and moderate-certainty evidence. Venlafaxine and flunarizine provide intermediate options, while valproic acid should be reserved for refractory cases given poor tolerability.

### Implications for clinical practice

Based on our findings, clinicians can inform patients that all evaluated preventive treatments significantly reduce vestibular migraine attacks, with no clear ineffective options among those studied. The choice between treatments should be individualized based on patient characteristics, comorbidities, and preferences.

For patients without contraindications who prioritize maximum efficacy, propranolol represents a reasonable first choice despite indirect evidence, particularly given its low cost and extensive safety data in migraine prevention. Patients with cardiovascular contraindications to beta-blockers should consider venlafaxine or galcanezumab as alternatives with robust efficacy.

For patients prioritizing tolerability or those who have failed traditional preventives, galcanezumab offers the best evidence quality and zero discontinuation rate in trials, though cost and access may be limiting factors. The 3–4 month trial period observed across studies should set appropriate expectations for assessing treatment response.

The modest effect of LcS suggests it could serve as adjunctive therapy or for patients with mild disease preferring non-pharmacological approaches. Valproic acid should be reserved for refractory cases given its 25% discontinuation rate, and requires careful consideration in women of childbearing potential.

Clinicians should note that the treatment effects observed (approximately 3–7 fewer attacks monthly) represent clinically meaningful improvements, as most patients experience 5–18 attacks monthly at baseline. The high placebo response emphasizes the importance of patient education and expectation management as components of comprehensive care.

### Strengths and limitations

#### Strengths

This network meta-analysis has several methodological strengths. First, we conducted comprehensive searches across multiple databases without language restrictions, supplemented by trial registry searches and reference screening. Second, we followed rigorous methodology including protocol registration, duplicate screening, and standardized data extraction. Third, this represents the first network meta-analysis to include CGRP monoclonal antibodies, providing timely evidence for emerging treatments. Fourth, we performed extensive sensitivity analyses confirming the robustness of our findings, with fixed and random-effects models yielding identical results. Fifth, we transparently reported evidence certainty using GRADE methodology, enabling appropriate interpretation of results. Finally, our threshold analysis quantified the stability of rankings, showing that substantial changes would be required to alter clinical conclusions.

#### Limitations

Several limitations warrant consideration. The star-shaped network structure prevented formal consistency testing, requiring assumptions about transitivity. Most treatment effects relied on indirect evidence, with only three treatments having direct placebo comparisons. Small sample sizes (17–103 per arm) resulted in wide confidence intervals, particularly for propranolol despite its top ranking. The negligible heterogeneity (τ²<0.001), while suggesting consistent effects, may reflect limited statistical power rather than true homogeneity.

Outcome measurement varied across studies, requiring harmonization assumptions that may have introduced bias. Two potentially important RCTs (Bayer 2019, [28] Maksoud Nassar 2023 [29]) could not be included due to incompatible data reporting, possibly affecting network completeness. The maximum follow-up was 24 weeks, preventing assessment of long-term efficacy or safety. Adverse event reporting was inconsistent, limiting comprehensive safety comparisons.

Patient-level characteristics were inadequately reported to enable subgroup analyses by disease severity, comorbid migraine, or previous treatment exposure. The predominance of female participants (60–94%) may limit generalizability to male patients. Publication bias assessment was limited by the small number of studies. Finally, each drug class was represented by single agents, preventing estimation of class effects.

Another limitation is that included studies applied different diagnostic criteria: five used Bárány Society/IHS criteria, while four relied on Neuhauser’s criteria. The latter are generally considered less rigorous, which may introduce bias. However, sensitivity analyses excluding these studies yielded consistent trends.

Although outside the scope of our objectives, the role of drug dosage and titration remains critical for optimizing treatment efficacy and tolerability. Future trials should systematically evaluate dose-response relationships.

#### Evidence beyond the network

Several treatments showed promise in studies that could not be included in our network meta-analysis. The Maksoud Nassar 2023 study [29] compared cinnarizine, propranolol, and topiramate in 45 VM patients. While it showed comparable efficacy across all three agents, it could not be included due to reporting proportional changes (43–55% reductions) rather than absolute monthly attack frequency, and lacking a control arm for network connection. These findings remain clinically relevant, supporting efficacy across multiple drug classes.

Additionally, preliminary evidence suggests potential roles for other treatments. Amitriptyline was used alongside other oral preventives in the Görür 2021 observational study, [31] reflecting real-world clinical practice. The same study [31] also evaluated botulinum toxin injections, which may represent a potential option for refractory cases, pending validation in larger RCTs. While these treatments could not be incorporated into our quantitative synthesis, they warrant consideration in comprehensive vestibular migraine management, particularly for patients who fail first-line therapies.

#### Research implications

This analysis identifies critical evidence gaps requiring urgent attention. Direct comparison trials between propranolol and galcanezumab would resolve the efficacy-evidence paradox. Larger trials with standardized outcomes (monthly vertigo days) and longer follow-up would improve precision and enable assessment of sustained efficacy. Including multiple agents per drug class would enable proper class effect estimation.

Future trials should prioritize head-to-head comparisons between the top-ranked treatments identified in this analysis. Specifically, a three-arm trial comparing propranolol, galcanezumab, and venlafaxine would efficiently address multiple evidence gaps. Standardized outcome measures are essential - we recommend monthly vertigo days as the primary outcome with DHI as a key secondary outcome. Trials should be powered for subgroup analyses by baseline vertigo frequency, comorbid migraine presence, and prior preventive exposure.

Long-term extension studies are needed to assess sustained efficacy beyond 6 months and capture delayed adverse events. Given the high placebo response observed, trial designs should consider run-in periods or enrichment strategies. Individual patient data meta-analysis would enable exploration of treatment effect modifiers and optimal patient selection.

Research into combination strategies is warranted, particularly adding LcS to conventional preventives given its excellent safety profile and potential complementary mechanism. The gut-brain axis represents an underexplored therapeutic avenue in vestibular migraine. Finally, biomarker studies could identify treatment response predictors, moving toward personalized therapy selection.

### What this study adds

#### What is already known on this topic

- Vestibular migraine affects 1–3% of the population and causes substantial disability.
- Various preventive medications are used based on migraine prophylaxis evidence.
- Previous systematic reviews evaluated only direct comparisons between individual treatments.

#### What this study adds

- First network meta-analysis comparing all available preventive treatments including CGRP antagonists.
- All evaluated treatments significantly reduce vertigo frequency (2.6–7.0.6.0 fewer attacks/month).
- Propranolol showed the largest effect but relies on indirect evidence; galcanezumab offers the best balance of efficacy and evidence quality.
- Treatment selection should consider the trade-off between statistical efficacy rankings and evidence certainty.

## Conclusions

All evaluated preventive treatments effectively reduce vertigo frequency in vestibular migraine, with effect sizes ranging from 2.6 to 7.0 fewer attacks per month. While propranolol showed the largest statistical effect, galcanezumab offers the best balance of efficacy, tolerability, and evidence quality. Treatment selection should consider individual patient factors, and combination approaches warrant investigation. Standardized trial methodology and head-to-head comparisons are urgently needed to optimize treatment selection for this disabling condition.

Article Highlights:

- All preventive treatments evaluated significantly reduce vestibular migraine attacks (2.6–7.0.6.0 fewer attacks/month).
- Propranolol ranked highest statistically but evidence is indirect; galcanezumab has the best quality evidence.
- Zero heterogeneity suggests consistent treatment effects across studies.
- Choice of treatment should balance efficacy rankings with evidence quality and individual patient factors.
- Head-to-head trials are urgently needed, particularly comparing propranolol with CGRP antagonists.

## Supplementary Information

Supplementary Material 1. Supplementary Table 1. Database Search Strategies and Results. Supplementary Table 2. Characteristics of Excluded Studies. Supplementary Table 3. Risk of Bias Detailed Assessment. Supplementary Table 4. GRADE Evidence Profiles. Supplementary Table 5. Sensitivity Analysis Results. Supplementary Figure 1. Risk of Bias Summary

Supplementary Material 2. Supplementary File 2. PRISMA-NMA Checklist

## Data Availability

Data is provided within the manuscript or supplementary information files.

## Abbreviations

VM: Vestibular migraine
CGRP: Calcitonin gene–related peptide
DHI: Dizziness Handicap Inventory
LcS: Lactobacillus casei Shirota
