# Supplementary material for: Comparative effectiveness and safety of preventive treatments for vestibular migraine: a systematic review and network meta-analysis
Source: BMC Neurol. 2025 Dec 30;25:513. doi: 10.1186/s12883-025-04490-0 (PMC12754936; doi:10.1186/s12883-025-04490-0)
Supplement: Supplementary file 1 — Supplementary Material 1. Supplementary Table 1. Database Search Strategies and Results. Supplementary Table 2. Characteristics of Excluded Studies. Supplementary Table 3. Risk of Bias Detailed Assessment. Supplementary Table 4. GRADE Evidence Profiles. Supplementary Table 5. Sensitivity Analysis Results. Supplementary Figure 1. Risk of Bias Summary [file 12883_2025_4490_MOESM1_ESM.docx]

# **Supplementary Materials**

## **Comparative Effectiveness and Safety of Preventive Treatments for Vestibular Migraine: A Systematic Review and Network Meta-Analysis**

## **Supplementary Table 1. Database Search Strategies and Results**

| **Database** | **Search Strategy** | **Date** | **Results** |
| --- | --- | --- | --- |
| **Embase (Ovid)** | (vestibular migraine or migrainous vertigo or migraine-associated vertigo).mp. and (prevention or prophylaxis or prophylactic or preventive).mp. and (randomized or randomised or RCT or placebo or trial or cohort or prospective).mp. | January 15, 2025 | 132 |
| **Scopus** | TITLE-ABS-KEY("vestibular migraine" OR "migrainous vertigo" OR "migraine-associated vertigo") AND TITLE-ABS-KEY(prevention OR prophylaxis OR prophylactic OR preventive) AND TITLE-ABS-KEY(randomized OR randomised OR RCT OR placebo OR trial OR cohort OR prospective) | January 15, 2025 | 101 |
| **PubMed** | ("vestibular migraine" OR "migrainous vertigo" OR "migraine-associated vertigo") AND (prevention OR prophylaxis OR prophylactic OR preventive) AND (randomized OR randomised OR RCT OR placebo OR trial OR cohort OR prospective) | January 15, 2025 | 75 |
| **Cochrane Library** | ("vestibular migraine" OR "migrainous vertigo" OR "migraine-associated vertigo") AND (prevention OR prophylaxis OR prophylactic OR preventive) | January 15, 2025 | 32 |
| **Total** |  |  | **340** |
| **After deduplication** |  |  | **178** |

### **Additional Sources Searched:**

- ClinicalTrials.gov: 48 records identified, 14 unique trials without published results
- WHO International Clinical Trials Registry Platform: 12 records identified, all duplicates
- Reference lists of included studies: 3 additional studies identified, all duplicates
- Conference abstracts (2023-2025): 4 identified, all preliminary results of included studies

## **Supplementary Table 2. Studies Excluded at Full-Text Review with Reasons (n=44)**

### **Studies with Detailed Review (n=25)**

| **Study** | **Primary Reason** | **Additional Reasons** |
| --- | --- | --- |
| Islam et al., 2023 | No control group | Duration <12 weeks (8 weeks) |
| Calandre et al., 2024 | Review article | Not primary study |
| Obermann & Strupp, 2014 | Review article | Not primary study |
| Kang et al., 2016 | Not intervention study | Prognostic study |
| Alghadir & Anwer, 2018 | Non-pharmacological | Vestibular rehabilitation |
| Hu et al., 2021 | Duration <12 weeks | Protocol only, 8-week duration |
| Hoskin & Fife, 2022 | Sample size <30 (n=28) | Retrospective design |
| Mallampalli et al., 2022 | Not primary study | Expert panel report |
| Oh et al., 2022 | Intervention not in protocol | Botulinum toxin, 8-week duration |
| Chu et al., 2023 | Network meta-analysis | Not primary study |
| Teelucksingh et al., 2023 | Review article | Pregnancy-specific |
| Hannigan et al., 2024 | No control group | Nutraceuticals not in protocol |
| Çelik et al., 2020 | No control group | Single-arm observational |
| Lovato et al., 2023 | Sample size <30 (n=23) | CGRP study, retrospective |
| Abu-Zaid et al., 2024 | Retrospective design | Supplements not in protocol |
| Karaaslan et al., 2020 | Not intervention study | Biomarker study |
| Çelebisoy et al., 2016 | Retrospective design | Used ICHD-3 beta criteria |
| Domínguez-Durán et al., 2020 | Duration <12 weeks | 5-week study, no control |
| Fotuhi et al., 2009 | Review article | Not primary study |
| Dieterich et al., 2016 | Review article | Not primary study |
| Teggi et al., 2015 | Intervention not in protocol | Fixed combination drug |
| Ceriani, 2024 | Review article | Not primary study |
| Beh et al., 2021 | Wrong population | MDDS not VM |
| Neuhauser et al., 2006 | Duration <12 weeks | 3-month open extension excluded |
| Yuan et al. 2016 | Sample size <30(23) | Prospective study, small sample size |

### **Summary of Exclusion Reasons (n=44)**

| **Exclusion Category** | **n (%)** |
| --- | --- |
| **Wrong study design** | 14 (31.8%) |
| - Review/commentary | 10 (22.7%) |
| - Protocol only | 2 (4.5%) |
| - Not intervention study | 2 (4.5%) |
| **No control group** | 8 (18.2%) |
| **Duration <12 weeks** | 7 (15.9%) |
| **Wrong intervention** | 6 (13.6%) |
| **Clinical trial registry (no results)** | 14 (31.8%) |
| **Conference abstract only** | 3 (6.8%) |
| **Retrospective design** | 4 (9.1%) |
| **Sample size <30 (CGRP)** | 3 (6.8%) |
| **Wrong population** | 1 (2.3%) |

*Note: Some studies had multiple exclusion reasons; primary reason listed

## **Supplementary Table 3. Risk of Bias Detailed Assessment**

### **Randomized Controlled Trials (Cochrane RoB 2.0)**

| **Study** | **D1: Randomization** | **D2: Deviations** | **D3: Missing Data** | **D4: Measurement** | **D5: Selection** | **Overall** |
| --- | --- | --- | --- | --- | --- | --- |
| **Sharon 2024** | Low | Low | Low | Low | Low | **Low** |
| - | Computer-generated, centralized | Double-blind maintained | 5% missing, balanced | Validated VM-PATHI | Pre-specified protocol |  |
| **Liu 2017** | Some concerns | High | Some concerns | Some concerns | Low | **High** |
| - | Quasi-randomization by admission | Single-blind, possible deviations | 13% missing, unclear handling | Self-reported diaries | All outcomes reported |  |
| **Salviz 2015** | Low | Some concerns | Low | Some concerns | Low | **Some concerns** |
| - | Random number table | Open-label design | ITT analysis, 19% dropout | Patient-reported, unblinded | Protocol registered |  |
| **Qi 2020** | Low | Low | Some concerns | Low | Low | **Some concerns** |
| - | Block randomization | Double-blind | 15% missing, per-protocol only | Standardized assessment | Pre-specified outcomes |  |
| **Lepcha 2014** | Low | Some concerns | Low | Some concerns | Low | **Some concerns** |
| - | Computer-generated | Open-label | Complete follow-up | Categorical outcomes only | All outcomes reported |  |
| **Bayer 2019** | Low | Low | High | Some concerns | Some concerns | **High** |
| - | Centralized IVRS | Double-blind | 37% dropout, early termination | Electronic diary | Terminated early |  |
| **Maksoud Nassar 2023** | Some concerns | High | Low | Some concerns | Low | **High** |
| - | Method not described | Open-label | No dropouts | Unblinded assessment | Complete reporting |  |

### **Observational Studies (ROBINS-I)**

| **Study** | **Confounding** | **Selection** | **Classification** | **Deviations** | **Missing** | **Measurement** | **Reporting** | **Overall** |
| --- | --- | --- | --- | --- | --- | --- | --- | --- |
| **Russo 2023** | Moderate | Low | Low | Low | Low | Moderate | Low | **Moderate** |
| - | Adjusted for baseline | Consecutive enrollment | Clear drug/dose | Protocol adherence | Complete data | Self-reported diary | All outcomes |  |
| **Görür 2021** | Serious | Low | Low | Moderate | Moderate | Moderate | Low | **Serious** |
| - | Non-randomized allocation | All eligible included | Treatment documented | Variable adherence | 18% dropout | Unblinded | Complete |  |

## **Supplementary Table 4. GRADE Evidence Profiles**

### **Primary Outcome: Monthly Vertigo Frequency Reduction**

| **Comparison** | **Studies (n)** | **Risk of Bias** | **Inconsistency** | **Indirectness** | **Imprecision** | **Other** | **MD (95% CI)** | **Certainty** |
| --- | --- | --- | --- | --- | --- | --- | --- | --- |
| **Galcanezumab vs Control** | 1 (40) | Not serious | Not assessable¹ | Not serious | Serious² | None | -5.80 (-10.61 to -0.99) | ⊕⊕⊕⊝ **Moderate** |
| **Propranolol vs Control** | 0 (0)³ | Serious⁴ | Not assessable¹ | Serious⁵ | Serious² | None | -7.04 (-12.77 to -1.31) | ⊕⊕⊝⊝ **Low** |
| **Venlafaxine vs Control** | 0 (0)³ | Serious⁴ | Not assessable¹ | Serious⁵ | Serious² | None | -5.94 (-8.98 to -2.90) | ⊕⊕⊝⊝ **Low** |
| **Flunarizine vs Control** | 1 (52) | Serious⁶ | Not assessable¹ | Not serious | Serious² | None | -4.00 (-6.54 to -1.46) | ⊕⊕⊝⊝ **Low** |
| **Valproic acid vs Control** | 0 (0)³ | Serious⁴ | Not assessable¹ | Serious⁵ | Very serious⁷ | None | -5.95 (-9.01 to -2.89) | ⊕⊝⊝⊝ **Very low** |
| **LcS vs Control** | 1 (247) | Serious⁶ | Not assessable¹ | Not serious | Not serious | None | -2.59 (-3.31 to -1.87) | ⊕⊕⊝⊝ **Low** |

¹Single study per comparison or no closed loops in network ²Wide confidence intervals and/or small sample size ³Indirect evidence only through network ⁴High risk of bias in contributing studies ⁵All evidence indirect through network connections ⁶Some concerns in risk of bias assessment ⁷Very wide confidence intervals, smallest sample size

### **Secondary Outcomes**

| **Outcome** | **Comparison** | **Studies** | **Participants** | **Effect Estimate** | **Certainty** |
| --- | --- | --- | --- | --- | --- |
| **DHI Score** | Galcanezumab vs Control | 1 | 40 | MD -13.7 (-20.4 to -8.5) | ⊕⊕⊕⊝ Moderate |
| **Responder Rate** | Galcanezumab vs Control | 1 | 40 | RR 1.60 (0.92 to 2.79) | ⊕⊕⊝⊝ Low |
| **Discontinuation** | All treatments | 7 | 419 | Range 0-25% | ⊕⊕⊝⊝ Low |

## **Supplementary Table 5. Sensitivity Analysis Results**

### **SA1: Fixed-Effect Model**

| **Treatment** | **Random-Effects MD** | **Fixed-Effect MD** | **Difference** | **Rank Change** |
| --- | --- | --- | --- | --- |
| Propranolol | -7.04 | -7.04 | 0.000 | 0 |
| Valproic acid | -5.95 | -5.95 | 0.000 | 0 |
| Venlafaxine | -5.94 | -5.94 | 0.000 | 0 |
| Galcanezumab | -5.80 | -5.80 | 0.000 | 0 |
| Flunarizine | -4.00 | -4.00 | 0.000 | 0 |
| LcS | -2.59 | -2.59 | 0.000 | 0 |

**Interpretation:** Negligible heterogeneity (τ²<0.001) resulted in identical fixed and random-effects estimates

### **SA2: Threshold Analysis**

| **Analysis** | **Current Value** | **Threshold** | **Change Needed** | **% Change** |
| --- | --- | --- | --- | --- |
| Propranolol to lose #1 rank | -7.04 | -5.95 | 1.09 | 15.5% |
| Galcanezumab to become #1 | -5.80 | -7.04 | 1.24 | 21.4% |
| Significance threshold (Propranolol) | -7.04 | -1.31 | 5.73 | 81.4% |
| Clinical threshold (2 attacks/month) | All treatments | >2.0 | 0.59-5.04 | Exceeded |

### **SA3: Feasibility of Planned Sensitivity Analyses**

| **Planned Analysis** | **Feasible** | **Result/Reason** |
| --- | --- | --- |
| Fixed-effect model | ✓ Yes | Completed - identical results |
| Include observational | ✓ Yes | Network disconnected (2 subnetworks) |
| Low RoB only | ✗ No | Only 1 study (network collapses) |
| ≥6 months duration | ✗ No | No studies met criteria |
| 3-month outcomes only | ✗ No | Would exclude key evidence |
| Drug class analysis | ✓ Yes | Each class n=1, no within-class comparison |

## **Supplementary Table 6. Network Connectivity Matrix**

|  | **Control** | **Propranolol** | **Valproic acid** | **Venlafaxine** | **Galcanezumab** | **Flunarizine** | **LcS** |
| --- | --- | --- | --- | --- | --- | --- | --- |
| **Control** | - | 0 | 0 | 0 | 1 (Sharon) | 1 (Lepcha) | 1 (Qi) |
| **Propranolol** | 0 | - | 0 | 1 (Salviz) | 0 | 0 | 0 |
| **Valproic acid** | 0 | 0 | - | 1 (Liu) | 0 | 0 | 0 |
| **Venlafaxine** | 0 | 1 | 1 | - | 0 | 1 (Liu) | 0 |
| **Galcanezumab** | 1 | 0 | 0 | 0 | - | 0 | 0 |
| **Flunarizine** | 1 | 0 | 0 | 1 | 0 | - | 0 |
| **LcS** | 1 | 0 | 0 | 0 | 0 | 0 | - |

Numbers indicate direct comparisons; study names in parentheses

## **Supplementary Table 7. Outcome Harmonization Details**

| **Study** | **Original Scale** | **Original Values** | **Converted to Monthly** | **Conversion Method** |
| --- | --- | --- | --- | --- |
| Sharon 2024 | Dizzy days/month | Direct | No conversion needed | - |
| Liu 2017 | Attacks/month | Direct | No conversion needed | - |
| Salviz 2015 | Attacks/month | Direct | No conversion needed | - |
| Qi 2020 | Episodes/month | Direct | No conversion needed | - |
| Lepcha 2014 | Categorical frequency | Low/High | Estimated means | Assumed distributions |
| Bayer 2019 | Attacks/30 days | IRR only | Could not convert | Excluded from NMA |
| Maksoud Nassar 2023 | Proportional change | -0.43 to -0.55 | Could not convert | Excluded from NMA |
| Russo 2023 | Vertigo days/month | Direct | No conversion needed | - |
| Görür 2021 | Attacks/3 months | 5.4±1.7 baseline | Divided by 3 | 1.8±0.57/month |

## **Supplementary Table 8. Sample Size and Power Considerations**

| **Comparison** | **Total N** | **Per Arm** | **Power for 3 attacks/month difference*** | **Actual Difference** | **Significant** |
| --- | --- | --- | --- | --- | --- |
| Galcanezumab vs Control | 40 | 18/22 | 45% | 5.80 | Yes |
| Flunarizine vs Control | 52 | 25/27 | 52% | 4.00 | Yes |
| LcS vs Control | 247 | 103/144 | 99% | 2.59 | Yes |
| Propranolol vs Venlafaxine | 64 | 33/31 | 58% | 1.10 | No |
| Flunarizine vs Venlafaxine | 45 | 22/23 | 42% | 1.94 | Yes |
| Valproic acid vs Venlafaxine | 43 | 20/23 | 40% | 0.01 | No |

*Assuming SD=3.5, α=0.05, two-tailed test

## **Supplementary Figure Legends**


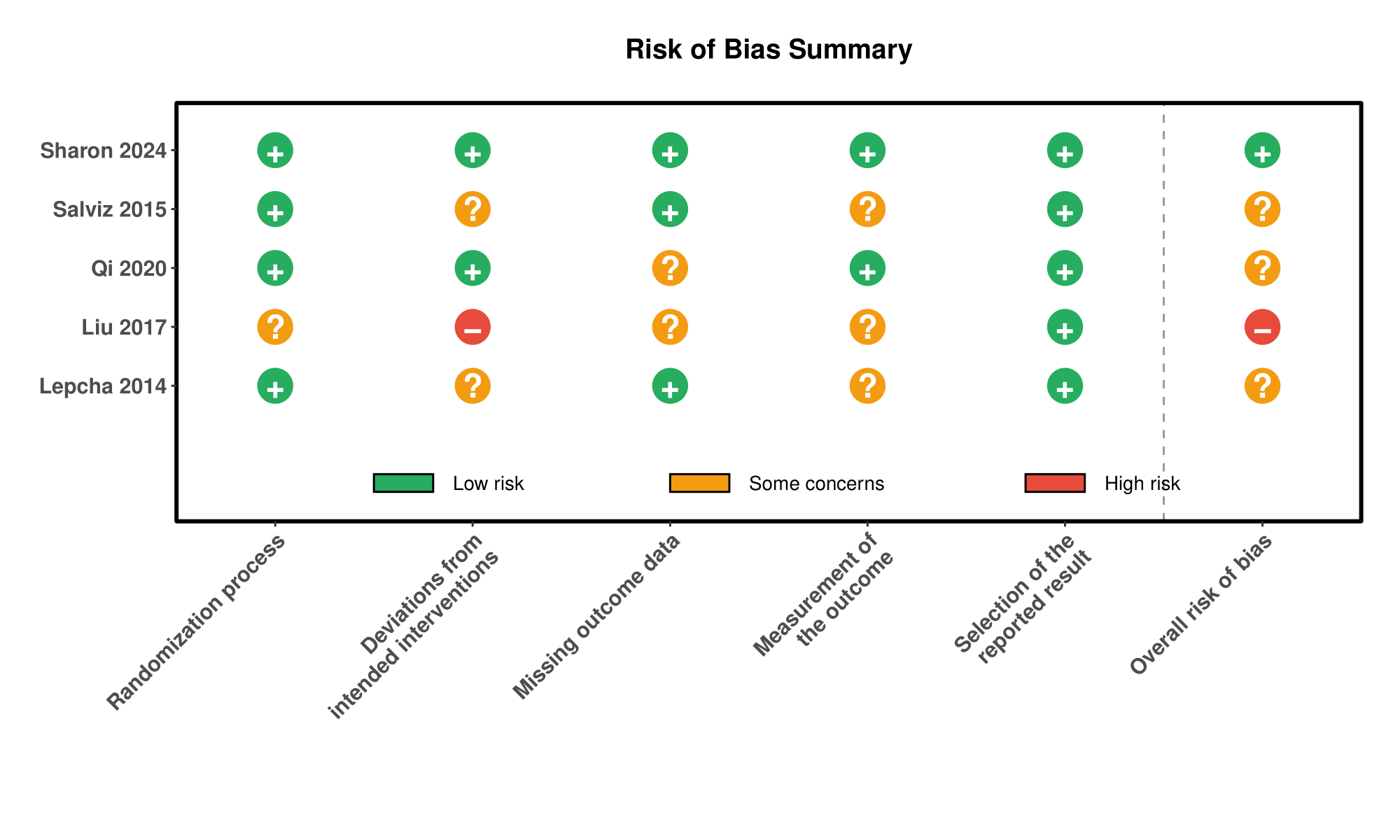


**Supplementary Figure 1. Risk of Bias Summary** Traffic light plot showing risk of bias assessments across five domains for each included randomized controlled trial. Green = low risk, yellow = some concerns, red = high risk.
